# Novel isochromosome 7q and *NOTCH1* mutation in an 88-year-old male with Sézary syndrome

**DOI:** 10.1016/j.jdcr.2025.09.030

**Published:** 2025-10-10

**Authors:** Nicole Loranger, Nicole Trepanowski, Jeremiah X. Karrs, Wahab A. Khan, Parth Shah, Joi B. Carter, Frederick Lansigan

**Affiliations:** a UMass Chan Medical School, Worcester, Massachusetts; b Department of Dermatology, Dartmouth-Hitchcock Medical Center, Lebanon, New Hampshire; c Department of Pathology and Laboratory Medicine, Dartmouth-Hitchcock Medical Center, Lebanon, New Hampshire; d Department of Hematology-Oncology, Dartmouth Cancer Center, Dartmouth-Hitchcock Medical Center, Lebanon, New Hampshire

**Keywords:** 7q, CTCL, cutaneous T-cell lymphoma, FISH, fluorescence in situ hybridization, genetic testing, genome, isochromosome, MF, mutation, mycosis fungoides, next generation sequencing, NGS, *NOTCH1*, SS, Sézary syndrome

## Introduction

Primary cutaneous T-cell lymphoma (CTCL) represents a heterogeneous collection of skin-limited lymphomas. Sézary syndrome (SS), an aggressive CTCL subtype, is unique for its comorbid leukemic and cutaneous involvement. Disease morphology varies, frequently mimicking other conditions and delaying diagnosis.^1^ Genetic analyses can provide insight into disease pathogenesis. We present an adult male with SS, found to have mutations not previously reported with this disease.

## Case report

**Fig 1:**
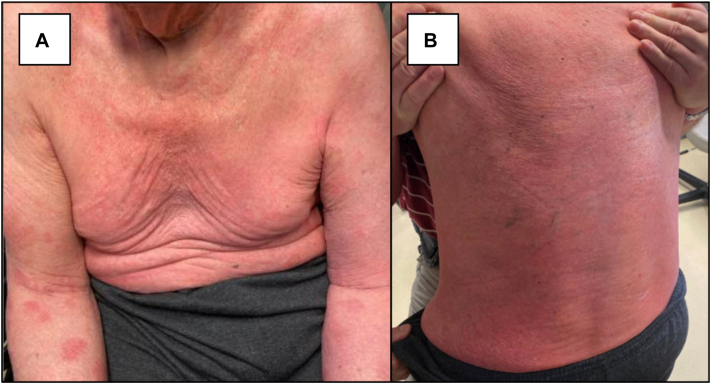
Sézary syndrome presenting as widespread, poorly demarcated, *pink* patches and plaques on the chest and upper extremities **(A)** and back **(B)** of an 88-year-old male.

**Fig 2:**
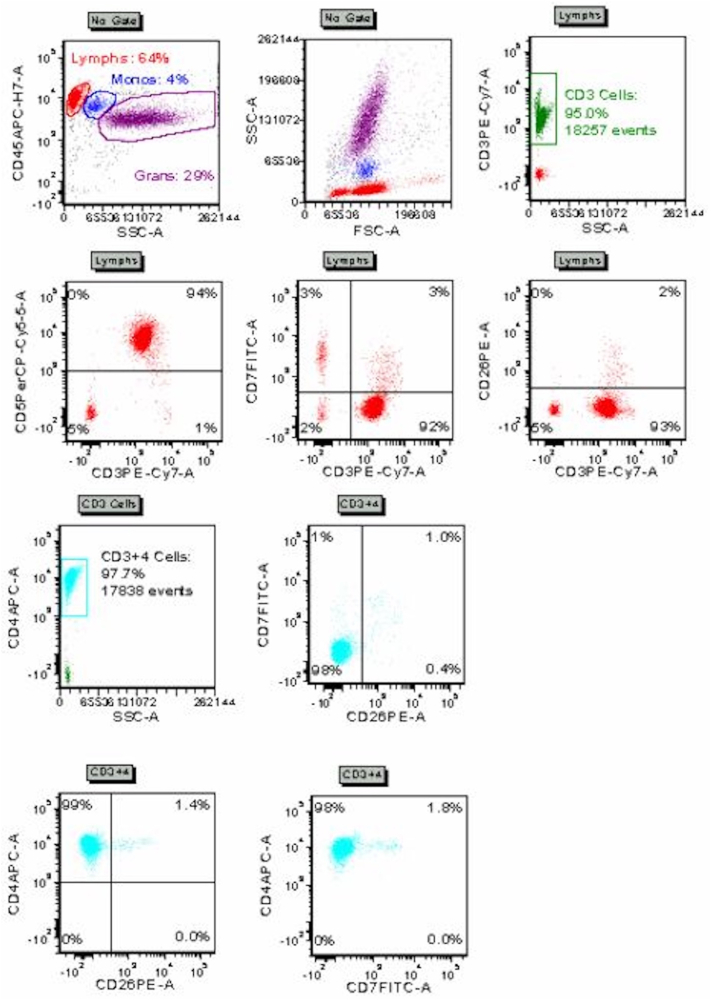
Flow cytometry demonstrating an aberrant T-cell population expressing CD4, CD2, CD5, TCR α/β and negative for CD7, CD26, CD10, TCR γ/δ.

**Fig 3:**
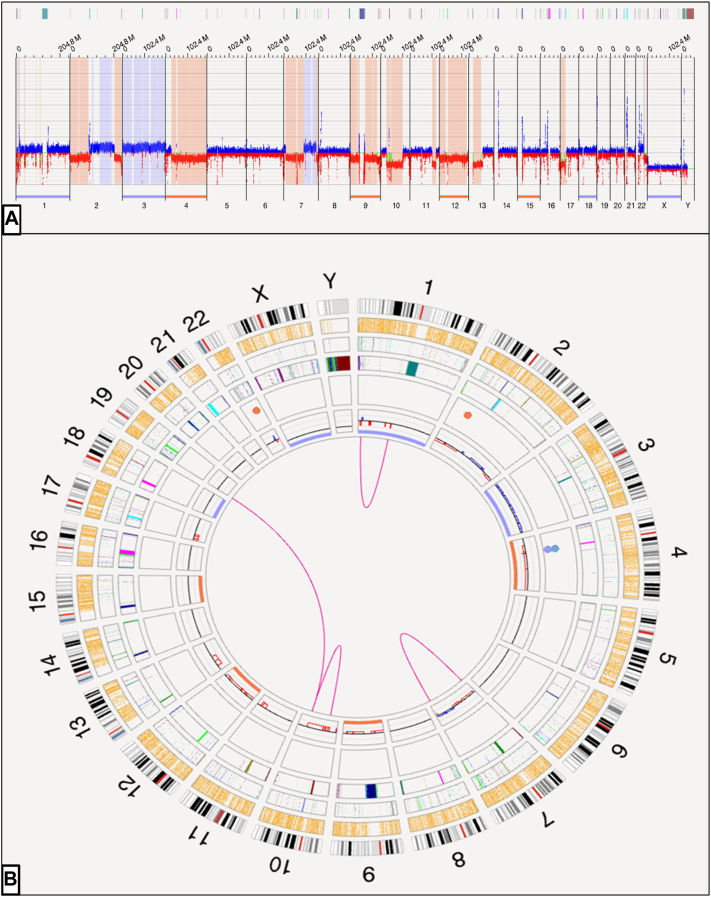
**A,** Whole genome view plot demonstrating copy number compared to a reference, with predominant blue and red signal indicating relative gains and losses, respectively. **B,** Optical genome mapping indicating translocations and inversions in the center (pink), relative copy number gains (blue) and losses (red) on inner ring, and deletions (orange), inversions (light blue), and duplications (light purple) on the fifth ring from the outside. Chromosomes are ordered sequentially and clockwise on the outermost ring, with centromeres labeled in red and cytological banding patterns denoted in gray scale.

An 88-year-old male presented to cutaneous lymphoma multidisciplinary clinic with 1 year history of asymptomatic full-body rash. Five months prior, he was hospitalized for a fall and incidentally noted to have widespread erythema, leukocytosis of 13.1 K/mcL, and peripheral smear with abnormal large cells suspicious for hematologic malignancy. At clinic presentation, he denied weight loss, fevers, or night sweats. Medications included atenolol 25 mg daily, hydrochlorothiazide 50 mg daily, vitamin C 500 mg daily, and vitamin B1 100 mg daily. Physical examination at clinic presentation revealed pink macules and papules coalescing into patches and plaques on the trunk and extremities and nummular, light pink, scaly plaques on the upper extremities, involving approximately 80% body surface area (Fig 1). Forearm and chest punch biopsies showed dense predominantly superficial dermal perivascular and interstitial infiltrate of atypical hyperchromatic lymphocytes with foci of epidermotropism, occasional Pautrier microabscesses, and background superficial dermal fibroplasia. Chest biopsy immunohistochemistry demonstrated atypical lymphocytes that were CD2+, CD3+, CD4+, CD5+, CD7-, TCR γ/δ-, with patchy expression of TCR α/β, and with CD4:CD8 ratio >10:1. Approximately 40% of lymphocytes expressed CD30. CD20 highlighted scattered background B cells. A monoclonal T-cell receptor rearrangement in TCR-β and TCR-γ was identified in skin using the LymphoTrack TRB/TRG Assay Panels. Peripheral blood flow cytometry identified lymphocytes with a CD4:CD8 ratio of 58.0, CD4+/CD7-population of 6202 lymphocytic cells/mcL (98.6%), CD4+/CD26-population of 6227 lymphocytic cells/mcL (97.8%), and monoclonal B-cell lymphocytosis (1.4% of lymphocytes) (Fig 2). Next-generation sequencing of peripheral blood revealed single nucleotide variation c.6577A>G p.Ser2193Gly in exon 34 of *NOTCH1* (variant allele frequency 16.8%). A peripheral blood T-cell lymphoma-focused fluorescence in-situ hybridization panel yielded extra copies of loci, suggestive of polyploidy. Additionally, a probe signal pattern within the 7q31/D7S486 locus showed extra copies of 7q in 49% of nuclei. Optical genome mapping (OGM) produced digital karyotype Circos and whole genome view plots (Fig 3). OGM detected gains of chromosomes 1, 2q11-q33.1, 3, 7q, 8q, 18, and 22q, and loss of chromosomes 2p, 2q33.3 (telomeric), 4, 7p indicating isochromosome 7q configuration, 9, 10q11.1q24.32, 11q23.3, 12, and 17p. An inversion disrupting *CHIC2* in proximity to *PDGFRA* was detected in 17% of cells with 29 molecule support calls, spanning the following breakpoints: ogm[GRCh38] inv(4) (q12q12) (54018499_54075983)[0.17]. Positron emission tomography-computed tomography demonstrated dermal activity in the flank and left buttock, diffuse fluorodeoxyglucose avidity in the right elbow soft tissue, multiple small mild-to-moderately fluorodeoxyglucose-avid lymph nodes in the bilateral axillary and inguinal regions, and a mildly fluorodeoxyglucose-avid 5.2 cm partially calcified cystic pancreatic tail mass. Pancreatic mass workup was deferred by patient. A diagnosis of SS was made.^1^ Given the patient was asymptomatic, home-based, and preferred less aggressive treatment with telehealth follow-up, he started bexarotene 150 mg daily. At 6 months telehealth follow-up, he had partial decrease in skin disease, not achieving 50% clearance, and met criteria for stable disease. By 10 months, patches and plaques were more confluent on the face and scalp, suggesting skin progression.

## Discussion

SS is a rare, aggressive subtype of CTCL. Diagnosis requires clinical erythroderma and evidence of peripheral blood involvement, including >1000 Sézary cells/μL, an expanded CD4+ T-cell population with a CD4:CD8 ratio >10:1, or an expanded T-cell population with an abnormal immunophenotype including >40% loss of CD7 or >30% loss of CD26.^1^

Genetic studies may provide insight into SS pathogenesis, aid in diagnosis and management, and predict outcomes. Current literature suggests SS has remarkable genetic heterogeneity, but some cytogenetic patterns have emerged. Mutations seen in our patient, including losses of chromosomes 2p (*DNMT3A*), 2q33.3 (telomeric; *PDCD1*), 4 (*VEGFC, FAT*), 7p (*DH11*, *ITGB8, EGFR*), 9 (*CDKN2A, PIP5K1B*), 10q11.1q24.32 (*FAS, PTEN, NFKB2*), 11q23.3 (*ATM*), 12 (*CDKN1B, NF1B*) and 17p (*TP53*, *TRIM16, RPA1, HIC1, MNT*), and gains of 1 (*TNFR2,TNFRSF1B, MYCL1*), 2q11-33.1 (*MAL*, *STAT4*), 3 (*RAF1, PIK3CA*), 4q12 (*CHIC2*), 7q (*BRAF*), 8q24.1 (*MYC*), 18 (*YES1*), and 22q (*BCR*) have been previously reported in SS.^2^, ^3^, ^4^, ^5^, ^6^ One study found MF/SS patients with *ATM* deletions had significantly lower overall survival than wild-type cases, whereas *MYC* amplification was not significantly associated with poorer overall survival, suggesting genetic testing may inform prognosis.^7^ A notable finding in our case is isochromosome 7q, a structural abnormality most commonly associated with hepatosplenic T-cell lymphoma and not previously reported in SS/CTCL to our knowledge. This mutation is believed to promote cell growth, proliferation, and drug resistance.^8^

An additional noteworthy finding in our case is the *NOTCH1* gene single nucleotide variation (9q34). Reports have described prominent *NOTCH1* expression in SS and increased apoptosis after targeting *NOTCH1* in SS cells.^9^ Although this variant has been rarely detected in population studies, the variant allele frequency of 16.8% observed here is significantly less than the 50% seen in non-mosaic germline mutations, suggesting a somatic variant, and its proximity to functional domains suggests a possible role in *NOTCH1* deregulation.^10^ No *NOTCH1* mutations have been reported in SS/CTCL to our knowledge; however, *NOTCH1* overexpression has been positively correlated with advanced MF/SS stage, suggesting a role for *NOTCH1* activity in SS/CTCL prognosis.^9^

We report novel mutations in *NOTCH1* and isochromosome 7q identified by fluorescence in-situ hybridization and OGM in the background of a polyploid clone in an SS patient. Despite initial stability in skin disease for 6 months on oral bexarotene, the patient ultimately progressed. The genetic alterations in this case as in others may be predictive of more resistant disease and deserves further study and follow-up. By reporting these findings, we aim to expand the growing collective understanding of SS, highlight the role of genetic assessments, and build momentum toward more targeted treatment recommendations for SS.

## Conflicts of interest

None disclosed.
